# Deletion of *Ptprd* and *Cdkn2a* cooperate to accelerate tumorigenesis

**DOI:** 10.18632/oncotarget.2106

**Published:** 2014-06-14

**Authors:** Berenice Ortiz, Julie R. White, Wei H. Wu, Timothy A. Chan

**Affiliations:** ^1^ Gerstner Sloan-Kettering Graduate School, Memorial Sloan-Kettering Cancer Center, New York, NY, USA; ^2^ Human Oncology and Pathogenesis Program, Memorial Sloan-Kettering Cancer Center, New York, NY, USA; ^3^ The Tri-Institutional Laboratory of Comparative Pathology, Memorial Sloan-Kettering Cancer Center, New York, NY, USA; ^4^ Dept. of Radiation Oncology, Memorial Sloan-Kettering Cancer Center, New York, New York, USA; ^5^ Brain Tumor Center, Memorial Sloan-Kettering Cancer Center, New York, New York, USA

**Keywords:** PTPRD, CDKN2A

## Abstract

*PTPRD* encodes the *protein tyrosine phosphatase receptor type D* and is frequently inactivated across many human cancers. Despite its frequent inactivation, it is unknown whether loss of *PTPRD* promotes tumorigenesis *in vivo*. *PTPRD* is located on chromosome 9p, as is *CDKN2A*, and the two loci are frequently deleted together. Here, we show that co-deletion of *Ptprd* and *Cdkn2a* cooperate to accelerate tumorigenesis. Interestingly, heterozygous loss of *Ptprd* was sufficient to promote tumorigenesis in our model, suggesting that *Ptprd* may be a haploinsufficient tumor suppressor. The loss of *Ptprd* resulted in changes to the tumor spectrum in mice and increased the frequency of lymphomas. In total, we reveal that *Ptprd* is a tumor suppressor that can promote tumorigenesis in concert with *Cdkn2a* loss.

## INTRODUCTION

*Protein tyrosine phosphatase receptor type D (PTPRD)* is a tumor suppressor gene on chromosome 9p. *PTPRD* inactivation is common in human malignancies and occurs in a number of cancer types including colorectal, esophageal adenocarcinoma, neuroblastoma, renal cell carcinoma, Ewing sarcoma, chronic myeloid leukemia, squamous cell carcinoma of the vulva, breast, lung cancer, melanoma, and glioblastoma [1,2,3,4,5,6,7,8,9,10,11]. Despite the high prevalence of *PTPRD* inactivation in human tumors, it is not known whether loss of *PTPRD* directly promotes tumorigenesis *in vivo*.

In humans, *PTPRD* is located on 9p23-24.3 and is telomeric to *CDKN2A*. The *CDKN2A* gene produces the p16^Ink4a^ and p14/p19^Arf^ tumor suppressors [12]. We and others have shown that selective pressure exists for inactivation of both genes on chromosome 9p, by deletion or mutation [8,10,11,13,14]. Despite the potential role of *PTPRD* loss in cancer, *Ptprd* deficient mice do not spontaneously develop tumors [15]. In contrast, 69% of *Cdkn2a−/−* mice develop tumors at an average age of 29 weeks [16]. We generated *Ptprd/Cdkn2a* co-deleted mice to determine if *Ptprd* loss contributes to tumorigenesis.

Here, we report that in the absence of *Cdkn2a*, *Ptprd* loss results in accelerated tumor development compared to mice lacking *Cdkn2a* alone. Both heterozygous and homozygous deletion of *Ptprd* accelerated tumorigenesis suggesting that loss of one copy of *Ptprd* is sufficient to act on tumor initiation or growth. Furthermore, loss of *Ptprd* changed the tumor spectrum, resulting in greater frequencies of aggressive lymphomas and histiocytic sarcomas. Our data show that *Ptprd* loss contributes to tumorigenesis in the setting of *Cdkn2a* deletion.

## RESULTS

### Genetic patterns of *PTPRD* loss in cancer

**Figure 1:**
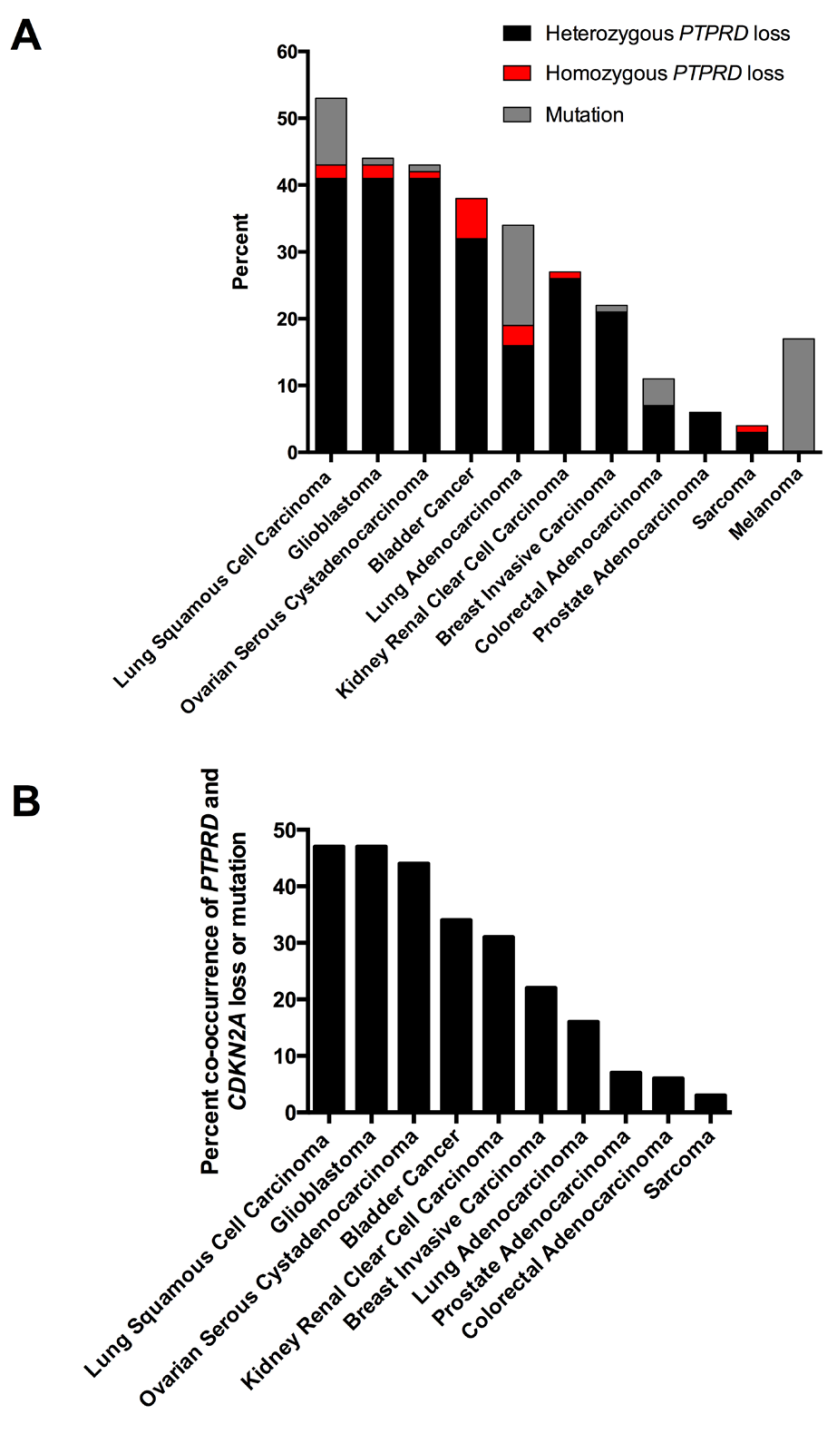
Genetic patterns of *PTPRD* loss in human cancers (A) Histogram of the frequency of mutation and copy number loss of *PTPRD* in human cancers. Point mutations, heterozygous loss, and homozygous deletions are presented. Data from the cBio Portal. (B) Histogram of the frequency of *PTPRD* and *CDKN2A* inactivation (mutation and deletion) in human cancers. Data from the cBio Portal.

We reviewed several genomic studies to define patterns of *PTPRD* loss in cancer. As shown in Figure 1A, *PTPRD* inactivation via deletion or mutation occurs frequently. In tumors with *PTPRD* copy number loss, loss of one copy of *PTPRD* occurs most commonly (Figure 1A). Moreover, co-deletion of *PTPRD* and *CDKN2A* occurs across a number of cancer types (Figure 1B). Co-occurrence of *PTPRD* and *CDKN2A* loss is significant across cancers (Figure 1B, *p*<0.05, 5 > Odds Ratio < 707, Table S1).

### *Ptprd* loss cooperates with *Cdkn2a* deletion to promote tumorigenesis

**Figure 2:**
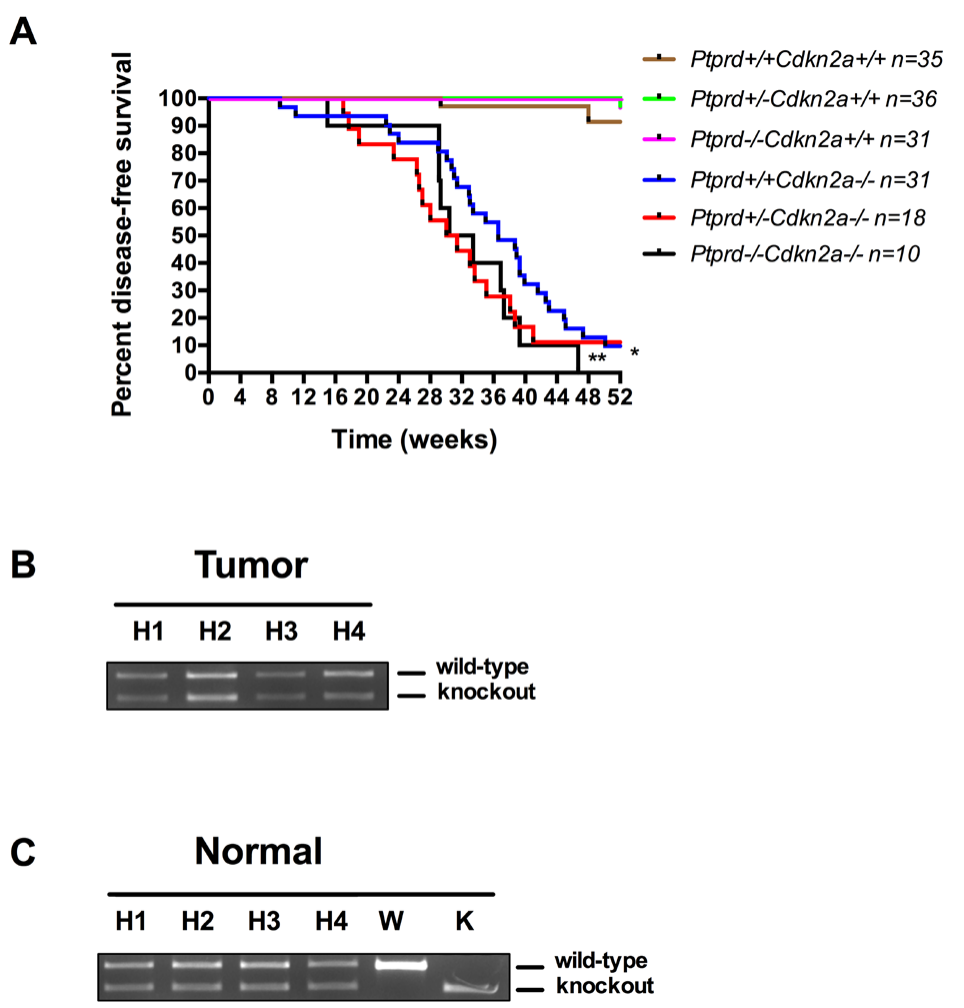
*Ptprd* loss cooperates with *Cdkn2a* deletion to promote tumorigenesis (A) Kaplan-Meier survival curve of *Ptprd+/+Cdkn2a+/+* (n=35), *Ptprd+/−Cdkn2a+/+* (n=36), *Ptprd−/−Cdkn2a+/+* (n=31), *Ptprd+/+Cdkn2a−/−* (n=31), *Ptprd+/−Cdkn2a−/−* (n=18), *and Ptprd−/−Cdkn2a−/−* (n=10) mice followed for 52 weeks. *Ptprd+/−Cdkn2a−/− vs. *Ptprd+/+Cdkn2a−/−* and ** *Ptprd−/−Cdkn2a−/−* vs. *Ptprd+/+Cdkn2a−/−*, p-value < 0.0001. (B) PCR genotyping of tumor tissue (histiocytic sarcoma) and (C) normal tissue for *Ptprd*. Tumors from *Ptprd+/−Cdkn2a−/−* mice retain an intact wild-type *Ptprd* allele. H = *Ptprd+/−Cdkn2a−/−*, W = *Ptprd+/+* control, K = *Ptprd−/−* control.

To investigate the role of *Ptprd* loss in tumorigenesis, we generated mice with loss of *Ptprd* alone, *Cdkn2a*, or both, and determined disease-free survival in each genotype. Mice were euthanized and necropsied at the time of onset of clinical signs (hunched, showing a swollen abdomen, or palpable lumps) or at a pre-determined endpoint. In accordance with Uetani et al. (2000), we did not observe tumor development in *Ptprd+/−* and *Ptprd−/−* mice (Figure 2A) [15]. However interestingly, *Ptprd+/−Cdkn2a−/−* and *Ptprd−/−Cdkn2a−/−* had significantly worse survival times than *Ptprd+/+Cdkn2a−/−* mice (Figure 2A, *p*<0.0001). These data suggest that *Ptprd* loss alone is not sufficient to initiate tumorigenesis but that in the context of *Cdkn2a* loss, *Ptprd* loss can cooperate to accelerate tumor development.

The results in Figure 2A suggest that loss of only one allele of *Ptprd* is sufficient to produce a phenotypic effect. In order to determine whether *Ptprd+/−* tumors retained an intact wild-type allele, we extracted DNA from tumors (histiocytic sarcoma) in *Ptprd+/−Cdkn2a−/−* mice and characterized *Ptprd* gene status using PCR. As shown in Figure 2B, tumors from *Ptprd+/−Cdkn2a−/−* mice retain an intact wild-type allele. As a control we extracted matched normal DNA (Figure 2C). Our results demonstrate that *Ptprd* loss and *Cdkn2a* deletion cooperate to promote tumorigenesis, and that heterozygous loss of *Ptprd* is sufficient to achieve this effect.

### Deletion of *Ptprd* and *Cdkn2a* alters the tumor spectrum

**Figure 3:**
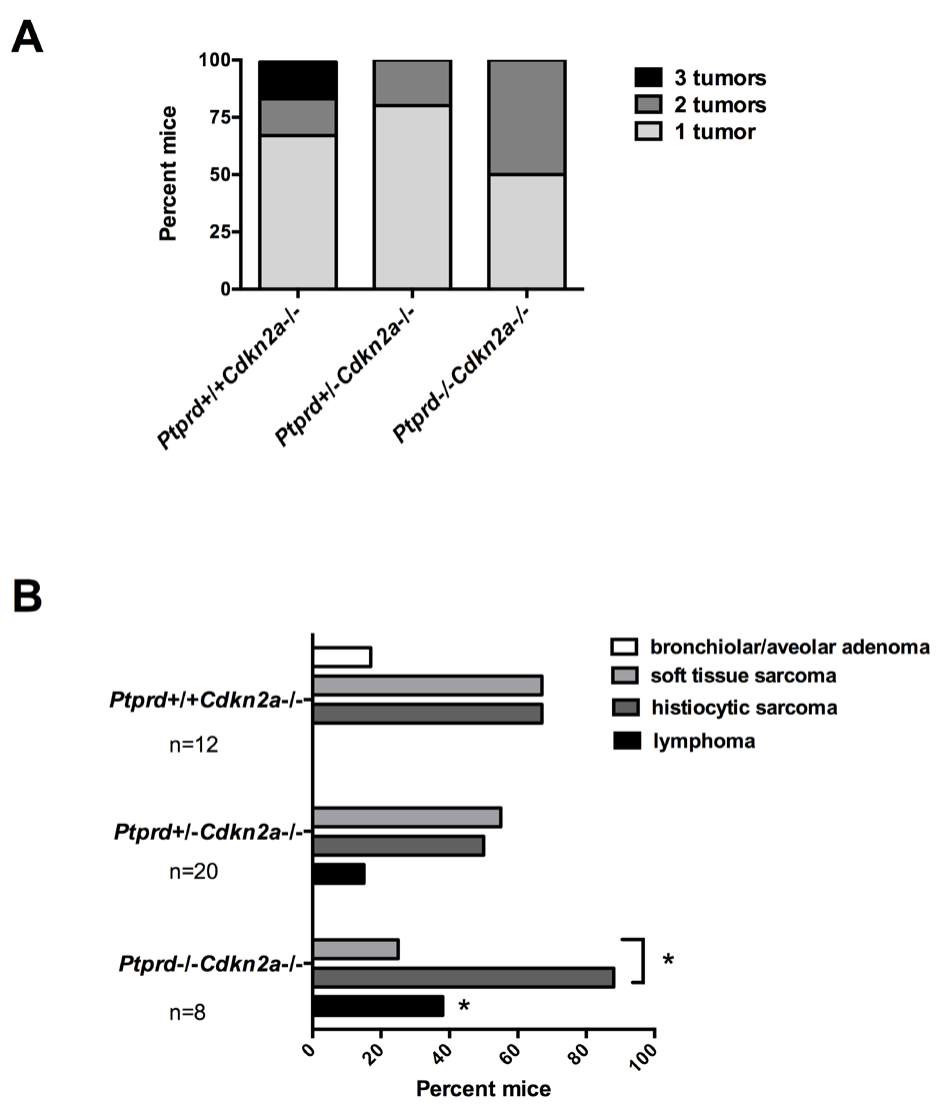
Mice with *Ptprd* loss and *Cdkn2a* deletion develop lymphomas, histiocytic sarcomas, and soft tissue sarcomas (A) Number of tumor types (lymphoma, histiocytic sarcoma, soft tissue sarcoma, or bronchiolar/alveolar carcinoma) per mouse of each genotype. (B) Frequency of tumor types by genotype. *Ptprd+/+Cdkn2a−/−* (n=12), *Ptprd+/−Cdkn2a−/−* (n=20), and *Ptprd−/−Cdkn2a−/−* (n=8) mice. *Ptprd−/−Cdkn2a−/− vs. *Ptprd+/+Cdkn2a9−/−* lymphomas p<0.05; *with bracket, *Ptprd−/−Cdkn2a−/−* histiocytic sarcomas vs. soft tissue sarcoma p<0.05.

In order to determine why mice with *Ptprd* loss had a faster onset of clinical signs, we first examined whether mice with *Ptprd* and *Cdkn2a* deletion had a greater number of tumor types compared to mice with *Cdkn2a* deletion alone. As shown in Figure 3A, no significant increases in the number of tumor types were observed in mice with *Ptprd* loss. In fact, mice with *Ptprd* loss tended to have fewer types of tumors.

**Figure 4:**
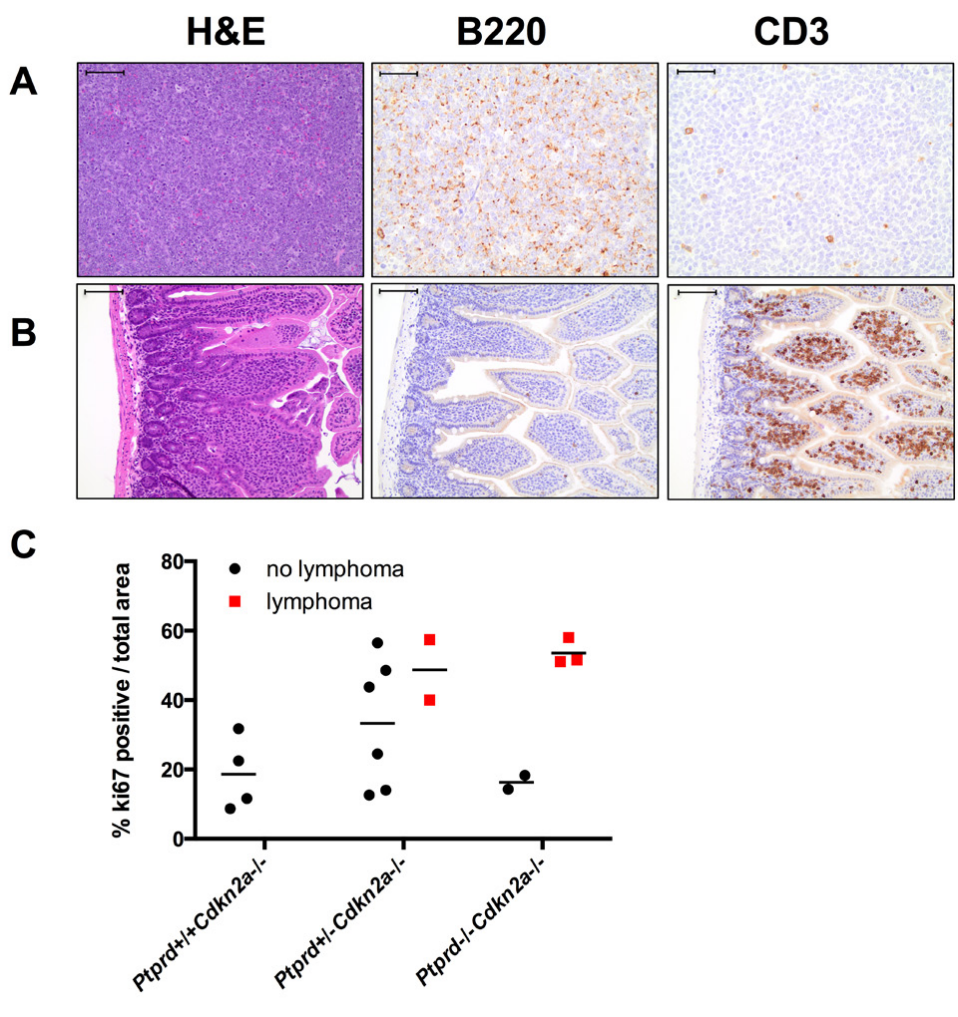
Lymphomas in mice with *Ptprd* and *Cdkn2a* loss (A) Representative images of *Ptprd+/−Cdkn2a−/−* B-cell lymphoma in a mesenteric lymph node. Left, scale bar = 100μm; Middle, B220 staining is used to identify B-cells, scale bar = 50μm; Right, CD3 staining is used to identify T-cells, scale bar = 50μm. (B) Representative images of *Ptprd−/−Cdkn2a−/−* T-cell lymphoma in the small intestine. Left, scale bar = 100μm; Middle, B220 staining, scale bar = 100μm; Right, CD3 staining, scale bar = 100μm. (C) Lymphomas in *Ptprd*+/−*Cdkn2a−/−* or *Ptprd−/−Cdkn2a−/−* mice have similar proliferative indices. Age-matched mesenteric lymph nodes with and without lymphoma were stained with Ki67 by immunohistochemistry.

We next determined whether *Ptprd* and *Cdkn2a* deletion altered the resultant tumor spectrum. Interestingly, *Ptprd−/−Cdkn2a−/−* mice developed significantly more lymphomas than *Ptprd+/+Cdkn2a−/−* mice (Figure 3B, *p*<0.05). In addition, *Ptprd+/−Cdkn2a−/−* mice showed a trend toward developing more lymphomas than *Ptprd+/+Cdkn2a−/−* mice (Figure 3B). Figure 4A and B shows examples of hematoxylin and eosin stained lymphomas in the mesenteric lymph node and small intestine, respectively. These tumors were composed of sheets of discrete round cells with scant basophilic cytoplasm and large round to polygonal nuclei. The neoplastic infiltrates often effaced normal tissue architecture, particularly within the lymph nodes (Figure 4A). In order to determine the cell origin of the lymphomas, we stained the tumors for B220, a B-cell marker, and CD3, a T-cell marker (Figure 4A, B). As listed in Table S2, all *Ptprd+/−Cdkn2a−/−* lymphomas were of a B-cell origin. Interestingly, 2/3 of the *Ptprd−/−Cdkn2a−/−* tumors were of T-cell origin (Table S2, Figure 4B). In order to quantitate the proliferative index of lymphoma cells, mesenteric lymph nodes from age-matched (28-39 weeks old) mice with or without lymphoma were stained for Ki67. Lymphomas from *Ptprd+/−Cdkn2a−/−* mice and *Ptprd−/−Cdkn2a−/−* mice had increased Ki67 staining, confirming the proliferative nature of the lymphomas (Figure 4C). Lymphomas from *Ptprd+/−Cdkn2a−/−* and *Ptprd−/−Cdkn2a−/−* mice had similar levels of Ki67 staining. Our results indicate that loss of *Ptprd* in *Cdkn2a* null mice promotes the development of lymphomas.

**Figure 5:**
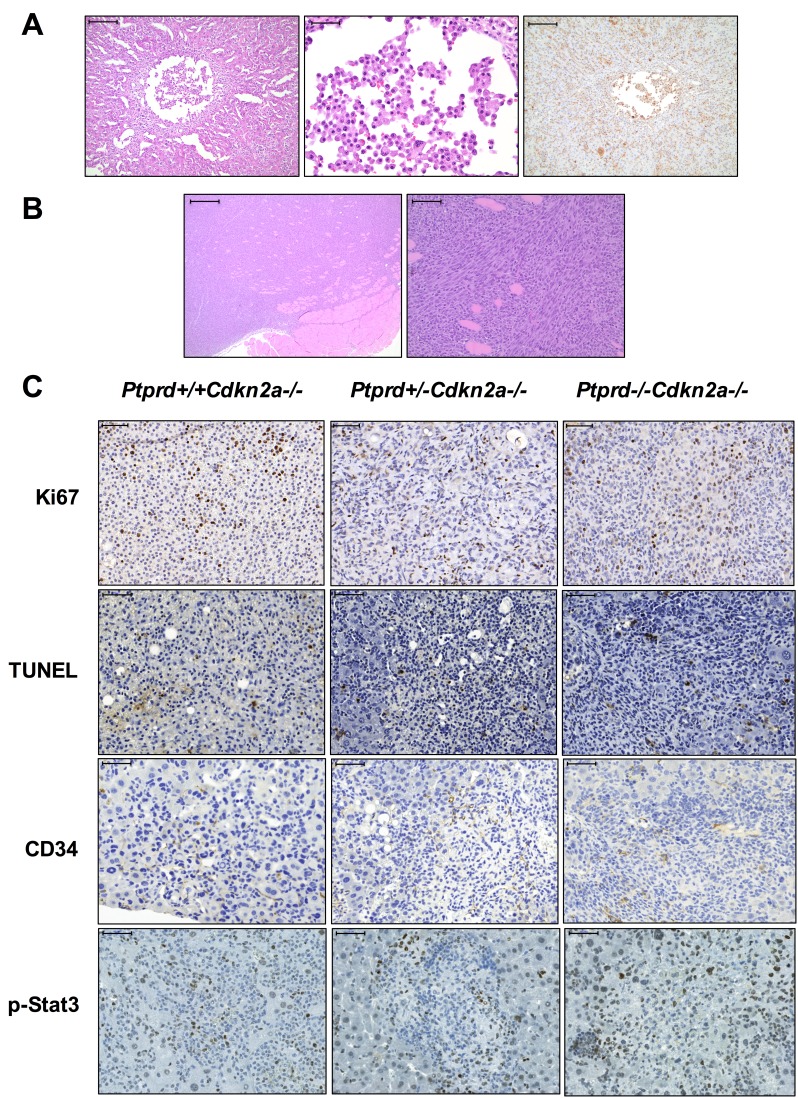
Histiocytic sarcomas and soft tissue sarcomas from mice with *Ptprd* and *Cdkn2a* deletion (A) Representative images of histiocytic sarcoma. Left, H&E staining of *Ptprd+/+Cdkn2a−/−* liver histiocytic sarcoma. Left, scale bar = 200μm; Middle, higher magnification of left image with scale bar = 50μm; Right, Mac-2 staining of *Ptprd+/+Cdkn2a−/−* liver histiocytic sarcoma with scale bar = 200μm. (B) Representative images of soft tissue sarcoma. Left, H&E staining of *Ptprd+/−Cdkn2a−/−* fibrosarcoma with scale bar = 500μm; Right, higher magnification of left image with scale bar = 100μm. (C) Representative images of *Ptprd+/+Cdkn2a−/−, Ptprd+/−Cdkn2a−/−*, and *Ptprd−/−Cdkn2a−/−* histiocytic sarcomas stained with Ki67, TUNEL, CD34, and p-Stat3. Scale bars = 50μm.

We observed that mice also developed either histiocytic sarcomas or soft tissue sarcomas (Table S2). Histiocytic sarcomas were composed of sheets of discrete round cells with moderate amounts of amphophilic cytoplasm and large round to polygonal nuclei. Within the liver, these cells frequently dissected between hepatic cords and regionally effaced normal tissue architecture (Figure 5A, *left*). Neoplastic cells were also frequently found within hepatic blood vessels (Figure 5A, *middle*). The cells stained positively with Mac2 consistent with histiocytic cell origin (Figure 5A, *right*). Soft tissue sarcomas (fibrosarcomas) were composed of streams, broad interlacing bundles, and frequent herringbone displays of spindle-shaped cells having moderate amounts of eosinophilic, fine fibrillar cytoplasm, poorly demarcated cell margins, and large oval to elongate nuclei. Within the skeletal muscle, these cells dissected between and frequently effaced myocytes (Figure 5B). While mice of all genotypes developed a similar frequency of sarcoma, it was interesting that *Ptprd−/−Cdkn2a−/−* mice developed significantly more histiocytic sarcomas than soft tissue sarcomas, suggesting that loss of both copies of *Ptprd* can preferentially promote the development of cancers with hematopoietic origin (Figure 3B, *p*<0.05).

We assessed levels of proliferation, apoptosis, and angiogenesis in the histiocytic and soft tissue sarcomas in *Cdkn2a*−/− mice with varying *Ptprd* genotype. All genotypes displayed positive staining for Ki67, TUNEL, and CD34, suggesting that loss of a single copy of *Ptprd* is sufficient to achieve tumor proliferation, apoptosis, and angiogenesis respectively (Figure 5C). However, no significant differences in the intensity or quantity of staining of Ki67, TUNEL, or CD34 were observed between the genotypes (Figure 5C, Table S3). Since it was previously shown that p-Stat3 is a substrate of PTPRD, we measured the levels of p-Stat3 by immunohistochemistry. No significant differences in the levels of p-Stat3 were observed, suggesting that Ptprd may have other substrates that mediate its tumor suppressive function in these tumor types (Figure 5C, Table S3).

## DISCUSSION

Our results highlight several important observations. First, we show that *Ptprd* loss promotes tumorigenesis in the setting of *Cdkn2a* deletion. Second, we show that heterozygous loss of *Ptprd* is sufficient to promote tumorigenesis. This is consistent with the hypothesis that the frequently observed heterozygous loss of *PTPRD* in human cancers contributes to tumor development. Third, our data suggests that loss of *Ptprd* plays a role in determining which types of tumors form.

Somatic loss of *PTPRD* has been associated with tumor risk and aggressiveness [4,11]. In addition, germ-line mutations of *PTPRD* are hypothesized to be involved in Ewing Sarcoma and glioblastoma [5,10]. We generated a mouse model with *Ptprd* and *Cdkn2a* loss in order to study the role of *Ptprd* in tumorigenesis. While mice with *Ptprd* loss alone did not show increased tumorigenesis, mice with *Ptprd* and *Cdkn2a* deletion had significantly shorter survival times than mice with *Cdkn2a* deletion alone. These results support the notion that there may be selective pressure for co-deletion of these two chromosome 9p tumor suppressors in human malignancies, and support a rationale for the patterns of *PTPRD* loss observed in human cancers. Although loss of *Ptprd* and *Cdkn2a* may be targeted in cancer, our data does not rule out the potential role of other tumor suppressors on chromosome 9p, including *Cdkn2b*.

In humans, loss of chromosome 9p occurs in B-cell lymphomas [26]. Intriguingly, in our study, mice with *Ptprd* loss developed more lymphomas. It has been shown that PTPRD is expressed in the B-cell lineage [27]. We speculate that the loss of *Ptprd* in our mice may alter B-cell biology, leading to of the development of lymphoma. It was also interesting that we observed a shift in the spectrum of sarcomas to a greater number of histiocytic sarcomas in *Ptprd−/−Cdkn2a−/−* mice. Histiocytes, or macrophages, are derived from circulating monocytes of bone marrow origin. Again, here, it would appear that *Ptprd* and *Cdkn2a* deletion increases the propensity of mice to develop tumors of hematopoietic origin.

In summary, we show that *Ptprd* copy number loss and *Cdkn2a* deletion cooperate to promote tumorigenesis. These findings have substantial implications for our understanding of a commonly inactivated tumor suppressor. Future studies will be needed to determine why *Ptprd* loss promotes tumorigenesis in some cell types but not others.

## METHODS

### Genetic Analysis of Human Tumors

The frequency of *PTPRD* inactivation and the co-occurrence of *PTPRD* and *CDKN2A* deletion were identified using genomic data in the cBio Portal [28].

### Generation of Mice

*Ptprd* heterozygous mice [15] were crossed to *Cdkn2a* knockout mice [16]. Mice were in a C57/Bl6 background. Mice were monitored twice a week and had complete necropsy performed if hunched, showing a swollen abdomen, or palpable lumps. Mice with *Cdkn2a* deletion were monitored until 52 weeks. Wild-type and mice with *Ptprd* loss alone were monitored for 104 weeks. Mice experiments were performed with MSKCC Institutional Animal Care and Use Committee approval.

### Histology and Pathology

The following tissues were dissected, fixed in 10% formalin (Sigma), and embedded in paraffin: heart, thymus, lung, tracheal/mandibular/mesenteric lymph nodes, kidneys, liver, pancreas, spleen, gall bladder, intestines, stomach, skin, urinary bladder, uterus/cervix/vagina/ovaries or testes/epdididymis/prostate/seminal vesicles, bone marrow, vertebral column, femur/tibia/surrounding muscles, sternum, eyes, tongue, teeth, salivary glands, adrenals, thyroid, esophagus, trachea, oral-nasal cavity, olfactory bulbs, brain, ear, pituitary, and thalamus. 5μM sections were stained with hematoxylin and eosin (H&E) and reviewed by a board-certified veterinary pathologist.

### Tumor Genotyping

Liver histiocytic sarcoma was macrodissected and DNA was extracted using the DNeasy Blood and Tissue kit (Qiagen). Tumors were confirmed by histological analysis. DNA from ear tissue was extracted as a normal tissue control. PCR was performed with the following *Ptprd* genotyping primers: 5′GGTGAAGTGTGACCAGTATTGGCC3′, 5′CTGGAATTGTCTCACTTTCCTC3′, and 5′GACTGCCTTGGGAAAAGCGCCTCC3′. Standard PCR procedures were performed with the following reaction buffer: 1M(NH_4_)_2_SO_4_, 2M Tris, pH 8.8, 1M MgCl_2_, and 14.4M B-mercaptoethanol.

### Immunohistochemistry

Dissected tissues were fixed in 10% formalin and embedded in paraffin. 5μM sections were used for immunohistochemical analysis. The Leica Bond RX automated system (Leica Biosystems) was used with the Polymer Refine Detection System (Leica Biosystems) for the following antibodies: B220 (BD Biosciences cat. no. 550286, mouse monoclonal, 1:200), CD3 (Vector, cat. no. VP-RM01, rabbit monoclonal, 1:100), Ki67 (Abcam, cat. no. ab16667, rabbit monoclonal, 1:100), and CD34 (Abcam, cat. no. ab8158, rat monoclonal, 16μg/mL). The Mac-2 (Cedarlane, cat.no. CL8942B, mouse monoclonal biotinylated) staining was performed manually with 10mM citrate retrieval for 30 minutes and the standard avidin/biotin immunoperoxidase protocol. p-Stat3 immunohistochemistry was performed using the Discovery XT processor (Ventana Medical Systems). Tissue sections were blocked in 10% goat serum with 2% BSA in PBS. Primary p-Stat3 Tyr-705 (Cell Signaling, cat no. 9145, rabbit monoclonal, 0.5μg/mL) antibody was incubated for 5 hours, followed by a 60 minute incubation with biotinylated goat anti-rabbit IgG (Vector Labs, cat. no. PK6101, 1:200 dilution) according to the manufacturer's instructions. Detection was performed with Blocker D, Streptavidin-HRP and DAB kit (Ventana Medical Systems) according to the manufacturer's instructions. Slides were counterstained with hematoxylin and coverslipped with Permount (Fisher Scientific). TUNEL staining was performed manually with the following reaction mixture: 0.1M Sodium Cacodylate pH7, 0.1mM DTT, 0.05mg/mL bovine serum albumin, 2u/ul terminal transferase, 0.2nm Biotin-16-dUTP, and 2.5mM Cobalt Chloride for 1 hour at 37 degrees. The reaction was terminated with 300mM sodium chloride and 30mM sodium citrate at room temperature for 15 minutes, incubated in avidin-biotin for 30 minutes, and developed with 3,3′-Diaminobenzidine for 3 minutes.

### Immunostaining Image Analysis

Whole slides were scanned with Pannoramic Flash Scanner (3DHistech, Hungary). Image analysis of tumor areas was performed with Metamorph software (Molecular Devices, PA). For analysis of immunohistochemistry images, color thresholds were set for brown positive staining and for total area (brown staining + blue nuclei).

### Statistical Analysis

Unless noted, student's t-test was performed for all statistical analysis. Log-rank statistical analysis was performed for Kaplan-Meier curves. Fisher's exact test was performed for the tumor spectrum analysis.

## SUPPLEMENTAL MATERIAL TABLES


