# Supplementary material for: Deletion of Ptprd and Cdkn2a cooperate to accelerate tumorigenesis
Source: Oncotarget. 2014 Jun 14;5(16):6976–82. doi: 10.18632/oncotarget.2106 (PMC4196177; doi:10.18632/oncotarget.2106)
Supplement: Supplementary file 1 [file oncotarget-05-6976-s001.pdf]

Deletion of *Ptprd* and *Cdkn2a* cooperate to accelerate tumorigenesis

Supplemental Material

Supplemental Table 1: Co-occurrence of *PTPRD* and *CDKN2A* inactivation in human cancers.

	***PTPRD* and *CDKN2A* inactivation**	**Total # of Samples**	**Percent co-occurrence**	**Odds Ratio**	**95% confidence interval**	**p-value**	**Study**
Clear Cell Renal Cell Carcinoma	129	418	31	707	237-2111	0	TCGA 2013 [17]
Ovarian Serous Cystadenocarcinoma	139	316	44	119	53-264	0	TCGA 2011 [18]
Glioblastoma	127	273	47	92	13-678	0	Brennan et al. 2013 [19]
Breast	105	482	22	88	45-170	0	TCGA 2012 [8]
Prostate	7	103	7	51	9-303	0.000003	Taylor et al. 2010 [20]
Colorectal Adenocarcinoma	12	212	6	34	10-117	0	TCGA 2012 [21]
Bladder	33	97	34	29	9-90	0	Iyer et al. 2013 [22]
Lung Squamous Cell Carcinoma	84	178	47	23	8-61	0	TCGA 2012 [23]
Sarcoma	7	207	3	16	4-66	0.0001	Barretina et. al 2010 [24]
Lung Adenocarcinoma	30	182	16	5	2-9	0.000007	Imielinski et al. 2012 [25]

Supplemental Table 2: Tumors in mice with *Ptprd* loss and *Cdkn2a* deletion.

Ptprd+/+Cdkn2a-/-				
**Mouse ID**	**Sex**	**Age**	**Diagnosis**	**Sites**
1	F	33	Soft Tissue Sarcoma	Abdominal mass
2	F	43	Histiocytic Sarcoma	Bone marrow, liver, lymph nodes (mesenteric), mesentery, ovaries, pancreas, small intestine, spleen, uterus
3	M	37	Histiocytic Sarcoma	Bone marrow, liver, spleen
4	M	11	Histiocytic Sarcoma	Spleen, lung
			Soft Tissue Sarcoma	Perirectal mass
			Bronchiolar/alveolar adenoma	Lung
5	M	45	Histiocytic Sarcoma	Bone marrow, brown fat, kidney, liver, lung, lymph nodes (mandibular, mesenteric, tracheobronchial), spleen, stomach, urinary bladder
			Soft Tissue Sarcoma	Liver
			Bronchiolar/alveolar carcinoma	Lung
6	F	43	Histiocytic Sarcoma	Duodendum, liver, lungs, lymph nodes, ovaries, oviduct, spleen, uterus
7	M	30	Histiocytic Sarcoma	Bone marrow, kidney, liver, lung, lymph nodes (mandibular, mesenteric), spleen, thymus
8	F	31	Soft Tissue Sarcoma	Flank skin
9	M	39	Histiocytic Sarcoma	Blood vessels (renal, meningeal, and brain), liver, lungs, lymph nodes, (mandibular, mediastinal, mesenteric), spleen, thymus
			Soft Tissue Sarcoma	Soft tissues of right head and neck
10	F	33	Histiocytic Sarcoma	Bone marrow, cecum, colon, connective tissue (mesenteric and paraovarian), lymph nodes (submandibular), ovaries, oviduct, thymus, uterus
			Soft Tissue Sarcoma	Axillary subcutis, connective tissue, lymph nodes (axillary), mammary gland, skeletal muscle, spinal cord/dura, vertebrae
11	M	22	Soft Tissue Sarcoma	Subcutis dorsal neck
12	M	33	Soft Tissue Sarcoma	Haired skin
Ptprd+/-Cdkn2a-/-				
13	F	27	Histiocytic Sarcoma	Bone marrow, liver, lymph node (mesenteric), oviducts

			Soft Tissue Sarcoma	Lower back mass
14	M	28	Histiocytic Sarcoma	Bone marrow, liver
			Soft Tissue Sarcoma	Vertebra, lungs, liver
15	F	33	Histiocytic Sarcoma	Lymph nodes (mandibular, mesenteric), spleen, thymus
16	M	17	Soft Tissue Sarcoma	Left flank mass
17	M	19	B-cell Lymphoma	Adipose tissue (perivetebral, periadrenal), eye, haired skin, kidney, liver, lungs, lymph nodes, parotid salivary gland, prostate gland, spleen, stomach, thymus
18	M	41	Histiocytic Sarcoma	Bone marrow, kidney, liver, lymph nodes (mesenteric), skin, spleen
19	M	28	B-cell Lymphoma	Liver, lymph nodes (mandibular, mediastinal, mesenteric), pancreas, spleen, subcutaneous tissues
20	F	34	Histiocytic Sarcoma	Oviduct, uterus
			B-cell Lymphoma	Lymph nodes (mandibular, mediastinal, mesenteric), oviduct, pancreas, spleen
21	M	18	Soft Tissue Sarcoma	Kidney, lumbar, lymph nodes (renal), pelvic mass
22	M	38	Histiocytic Sarcoma	Bone marrow, liver, lymph nodes, spleen
23	F	28	Soft Tissue Sarcoma	Leg mass
24	M	21	Soft Tissue Sarcoma	Left shoulder mass, lymph nodes
25	M	31	Histiocytic Sarcoma	Adipose tissue (parasternal, kidneys, liver, lungs, lymph nodes (mandibular, mesenteric), spleen
			Soft Tissue Sarcoma	Left thigh mass
26	F	39	Histiocytic Sarcoma	Bone marrow, cervix, liver, lymph nodes (mesenteric), ovaries, thymus, uterus, vagina
27	M	30	Histiocytic Sarcoma	Bone marrow, liver, lung, lymph nodes (mandibular, mesenteric), spleen, thymus
28	M	30	Histiocytic Sarcoma	Liver, lung, lymph nodes (tracheobronchial), spleen, thymus
29	M	28	Soft Tissue Sarcoma	Subcutaneous abdominal mass
30	M	6	Soft Tissue Sarcoma	Retobullar / subcutaneous mass
31	M	19	Soft Tissue Sarcoma	Left flank mass
32	M	31	Soft Tissue Sarcoma	Sublumbar soft tissues
Ptprd-/-Cdkn2a-/-				
33	F	37	Histiocytic Sarcoma	Liver, lymph nodes (mesenteric, pancreatic), ovaries, pancreas, stomach, uterus
			T-cell Lymphoma	Muzzle (skeletal muscle, bone subcutis)
34	F	39	Histiocytic Sarcoma	Bone marrow, blood vessels, liver, lungs, lymph nodes (mandibular, mesenteric, tracheobronchial), mesovarium, ovary, spleen, uterus

35	M	37	Histiocytic Sarcoma	Adipose tissue (brain, pervetrebral), blood vessels of kidneys, liver, lungs, mediastinal tissue/thymus
36	F	33	Histiocytic Sarcoma	Broad ligament of mesovarium and uterus, kidney, liver, lymph nodes (pancreatic and renal), ovaries, pancreas, spleen
37	F	39	Histiocytic Sarcoma	Bone marrow, kidneys, liver, lungs, lymph nodes (inguinal, mandibular, mesenteric), ovaries, small intestine, sternum, stomach, uterus
			T-cell Lymphoma	Small intestine, spleen
38	M	29	Soft Tissue Sarcoma	Thoracic mass
39	F	29	Histiocytic Sarcoma	Adipose tissue (mesenteric), adrenal glands, bone marrow, kidneys, liver, ovaries, pancreas, small intestine, spleen, stomach, uterus
			B-cell Lymphoma	Lungs, lymph nodes (axillary, inguinal, mandibular), thymus
40	F	47	Histiocytic Sarcoma	Bone marrow, intestines, liver, mesentery, ovaries, pancreas, urinary bladder, uterus
			Soft Tissue Sarcoma	Right stifle

Supplemental Table 3: Quantification of immunohistochemistry analysis of histiocytic sarcoma tumors in mice with *Ptprd* loss and *Cdkn2a* deletion. Values shown are % positive / total area ± standard deviation. No significant differences were observed between genotypes.

IHC Marker	*Ptprd+/+Cdkn2a-/-*	*Ptprd+/-Cdkn2a-/-*	*Ptprd-/-Cdkn2a-/-*
Ki67	32.7 ± 4.7	38.3 ± 4.7	34.2 ± 5.3
CD34	6.0 ± 5.1	6.1 ± 1.7	7.3 ± 0.9
TUNEL	2.6 ± 1.4	4.5 ± 1.5	8.4 ± 8.6
p-Stat3	29.4 ± 2.3	29.8 ± 26.2	23.5 ± 7.4
